# Prediction and Visual Analysis of Food Safety Risk Based on TabNet-GRA

**DOI:** 10.3390/foods12163113

**Published:** 2023-08-18

**Authors:** Yi Chen, Hanqiang Li, Haifeng Dou, Hong Wen, Yu Dong

**Affiliations:** 1 Beijing Key Laboratory of Big Data Technology for Food Safety, Beijing Technology and Business University, Beijing 100048, China; 2130072021@st.btbu.edu.cn (H.L.); dhaifeng@yeah.net (H.D.); 2 Hubei Provincial Institute for Food Supervision and Test, Wuhan 430075, China; hbsjyqt@163.com; 3 School of Computer Science, University of Technology Sydney, Sydney, NSW 2008, Australia; yu.dong-3@student.uts.edu.au

**Keywords:** food safety, risk prediction, visual analysis, early warning, TabNet, grey relational analysis

## Abstract

Food safety risk prediction is crucial for timely hazard detection and effective control. This study proposes a novel risk prediction method for food safety called TabNet-GRA, which combines a specialized deep learning architecture for tabular data (TabNet) with a grey relational analysis (GRA) to predict food safety risk. Initially, this study employed a GRA to derive comprehensive risk values from fused detection data. Subsequently, a food safety risk prediction model was constructed based on TabNet, and training was performed using the detection data as inputs and the comprehensive risk values calculated via the GRA as the expected outputs. Comparative experiments with six typical models demonstrated the superior fitting ability of the TabNet-based prediction model. Moreover, a food safety risk prediction and visualization system (FSRvis system) was designed and implemented based on TabNet-GRA to facilitate risk prediction and visual analysis. A case study in which our method was applied to a dataset of cooked meat products from a Chinese province further validated the effectiveness of the TabNet-GRA method and the FSRvis system. The method can be applied to targeted risk assessment, hazard identification, and early warning systems to strengthen decision making and safeguard public health by proactively addressing food safety risks.

## 1. Introduction

Food safety has emerged as a significant global public health concern in recent years, impacting people’s health and wellbeing [1]. Within the food supply chain, food products are susceptible to contamination due to various safety hazards, including biological, chemical, and physical risks [2]. These hazards can give rise to over 200 different diseases, ranging from mild conditions such as diarrhea to more severe outcomes such as cancer. Alarming data from the World Health Organization in 2023 indicate that over 600 million cases of foodborne illnesses and 420,000 deaths may result from consuming contaminated food annually [3]. This pressing situation underscores the urgent need to strengthen food safety supervision and thereby prevent incidents and safeguard public health. Monitoring potential hazards in food, conducting food safety risk predictions, and issuing early warnings have proven to be effective tools in the supervision and control of food safety. Food safety prediction involves utilizing models to predict future food safety events or outcomes by analyzing patterns from historical food-safety-related data to provide a basis for risk warnings. Such risk prediction is highly valuable in developing food safety surveillance programs, especially in identifying products and hazards that warrant close monitoring [4]. Consequently, the establishments of robust food safety risk prediction models hold crucial significance for both risk monitoring and early warning in the context of food safety.

Machine learning (ML), the process through which computers learn from substantial historical data via statistical algorithms, generate empirical models, and make predictions or decisions [5], has emerged as an effective approach to solving food safety risk prediction challenges in recent years [2]. The European Union launched the Rapid Alert System for Food and Feed (RASFF) portal in 1977 to ensure cross-border monitoring and a quick reaction when public health risks are detected in the food chain. In recent years, extensive research has been conducted on applying ML within the RASFF framework [2,6,7]. In 2014, the European Food Safety Authority (EFSA) assessed the potential of applying machine learning techniques (MLTs) to food risk assessment. Five case studies have been proposed based on data from the European Union Summary Reports on Zoonoses and on Antimicrobial Resistance. 

Random forests, clustering methods, and ensemble models have been investigated, and specific strategies, such as cross-validation, have been used to address well-known issues such as over-fitting [8]. For instance, Liu et al. [9] employed random forest (RF) classification to predict food non-conformity indicators, while Gao et al. [10] constructed a LightGBM risk warning model using integrated fuzzy hierarchical partitioning based on gradient boosting decision trees (GBDTs) to predict meat product safety risks. Wang et al. [11] utilized an extreme gradient boosting tree (XGBoost) to develop a prediction model for rice safety risks. These models integrate multiple decision tree models and fuse the results of various single models in different ways, effectively reducing any prediction bias associated with individual models and improving overall fitting ability. However, tree models are susceptible to data noise interference and tend to overfit when the tree depth is excessive, leading to inaccurate prediction results. 

On the other hand, artificial neural networks (ANNs) are increasingly used to solve classification and regression prediction problems due to their ability to learn more complex data patterns. For instance, neural networks such as extreme learning machines (ELMs) [12], radial basis functions (RBFs) [13,14], and backpropagation (BP) neural networks [15] have been utilized to construct efficient food safety risk prediction models for dairy products, meat products, and vegetables. Unlike tree models, ANNs possess end-to-end learning capability, eliminating the need for users to focus extensively on internal network processes. They can aptly approximate complex non-linear relationships, enabling them to more effectively learn patterns in intricate data, and they exhibit superior generalization ability. However, ANNs have certain limitations, such as their slow convergence and susceptibility to local optimization during the training process [16]. 

TabNet is a highly efficient standard deep neural network architecture designed specifically for tabular data [17]. It employs sequential attention to select salient features at each decision step, enabling more accurate and efficient learning. Combining the advantages of multiple decision-making steps in a tree model with the end-to-end learning ability of a neural network, TabNet exhibits robust fitting ability in tasks such as the classification and regression of tabular data. It addresses the issues encountered when using tree models, such as data noise interference leading to overfitting, and those affecting complex neural network structures, such as susceptibility to local optimization. TabNet has found applications in various fields, including rainfall prediction [18] and soybean protein P-site classification prediction [19]. This study endeavors to construct a TabNet-based food safety risk prediction model for the first time.

When developing a food safety risk prediction model, it is essential to analyze detection data and obtain comprehensive risk values in order to train the model. The analytic hierarchy process (AHP) is often employed for this purpose, but it is limited by its subjective weight-assignment method. Grey relation analysis (GRA), an important multivariate analysis method and part of grey system theory [20], measures the correlation between two objects based on the similarity of data geometry presented by sequence curves [21]. GRA can partially overcome the subjectivity issues present in methods such as AHP. In the field of food science, GRA has been utilized to calculate the correlation between food safety influences, determine the weight of each risk indicator, and fuse detection data to obtain comprehensive risk values [22,23]. Therefore, this study adopts the GRA method to derive comprehensive risk values for testing data.

Visualization techniques are widely recognized as an effective means for analyzing and interpreting data [24,25]. Intelligent systems that incorporate visualization techniques provide analysts with direct and efficient tools for exploring and interpreting data [26,27], significantly enhancing the efficiency of risk analysis and decision support for food safety regulations [28,29]. Furthermore, visualization techniques have found applications in other food-safety-related fields [30,31,32]. This study has designed and implemented the FSRvis system, a food safety risk prediction and visualization system that combines the TabNet-GRA method with advanced visual analysis techniques, including multi-view collaboration and human–computer interaction. This visualization system supports food safety risk prediction and interactive visual analysis based on detection data, offering valuable assistance to food safety supervision departments in conducting risk analysis and prediction.

The primary contributions of this work are as follows: (1) a novel risk prediction method for food safety, TabNet-GRA, which enables a rapid and precise determination of fine-grained risk values for food samples based on detection data by combining the advantages of TabNet and GRA, (2) a food safety risk prediction and visualization system, called FSRvis system, in conjunction with the TabNet-GRA method, which can facilitate food safety risk prediction and interactive visual analysis based on detection data, and (3) a case study employing detection data from cooked meat products in a Chinese province, which the results of validate the effectiveness of the TabNet-GRA method and the FSRvis system.

## 2. The Framework of TabNet-GRA Method and FSRvis System

**Figure 1:**
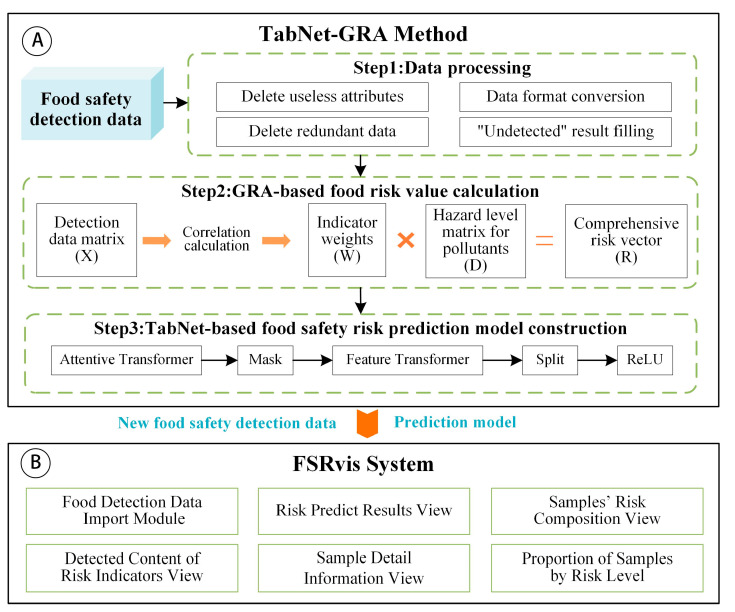
The framework of TabNet-GRA method and FSRvis system. (**A**) The pipeline of TabNet-GRA method; (**B**) the framework of FSRvis system.

The framework of the TabNet-GRA method and FSRvis system is illustrated in Figure 1. In Figure 1A, the progression of the food safety prediction method grounded in TabNet-GRA is delineated, while Figure 1B outlines the framework of the FSRvis system. The food safety risk prediction model, developed using the TabNet-GRA method, will be seamlessly integrated into the FSRvis system, facilitating food risk prediction. For more comprehensive understanding, readers are directed to consult Section 3 and Section 5.

## 3. The TabNet-GRA Method

TabNet-GRA is considered a food safety risk prediction method that utilizes the combination of TabNet and GRA, which can predict the risk of food products by employing food safety detection data. In this section, the principles and construction process of TabNet-GRA will be introduced.

### 3.1. The Pipeline of TabNet-GRA Method

The Pipeline of the TabNet-GRA method is illustrated in Figure 1A, encompassing the subsequent steps.

Step 1: Data processing. The food safety detection data are processed for deleting useless attributes, data format conversion, deleting redundant data and “undetected” result filling, etc., and the data are processed into a data matrix X suitable for modeling.

Step 2: Using GRA to calculate the comprehensive risk value of each food sample in a detection dataset. Firstly, GRA is used to calculate the weight vector W of each indicator’s contribution to the risk of the sample; secondly, the hazard level matrix for pollutants D is obtained using the detection value of the risk indicator compared with its corresponding limit value; and, finally, D is multiplied with W to obtain the risk vector of the food sample R.

Step 3: Construction and training of a TabNet-based food safety risk prediction model. The predictive model was constructed based on the TabNet. During the training process of the model, the food safety detection data matrix X is used as the input, and the risk vector of the food sample R is used as the expected output; the relevant parameters of the model are set and adjusted to obtain the TabNet-based food safety risk prediction model. The performance of the model is evaluated eventually.

### 3.2. The GRA-Based Food Risk Quantitative Assessment

Grey relational analysis (GRA) is a multi-indicator decision-making evaluation method developed from the gray system theory. Its fundamental concept involves quantifying the geometric similarity between reference data sequences and multiple comparative data sequences to establish their level of association. This analytical approach enables the assessment and evaluation of correlations and influences among multiple indicators, facilitating comprehensive decision-making progress [19]. In the second step of the TabNet-GRA method, GRA is utilized to determine the contribution weight of each indicator to the sample’s risk, and then the comprehensive risk value for each sample is calculated.

In order to clearly describe the process of calculating the sample’s comprehensive risk value, the definitions of the symbols used in it are first stated. Let X be the food detection data matrix, containing m indicators and n samples. xi(k) is the detection result of the ith indicator of the kth sample, where i=1,2,…,m;k=1,2,…,n, n is the length of the data sequence (the number of food samples), and m is the number of indicators. The reference sequence is $X_{1}=\{x_{1}(1),x_{1}(2),\ldots ,x_{1}(k),\ldots ,x_{1}(n)\}$ and the comparison sequence is $X_{i}=\{x_{i}(1),x_{i}(2),\ldots ,x_{i}(k),\ldots ,x_{i}(n)\}$. In the actual calculation, each indicator sequence is used as a reference sequence once, and the rest of the indicator sequences are used as a comparison sequence.

$$X=\left[\begin{array}{c} X_{1}\\ X_{2}\\ \vdots \\ X_{i}\\ \vdots \\ X_{m} \end{array}\right]={\left[\begin{array}{cccc} \begin{array}{c} x_{1}(1),x_{1}(2)\\ x_{2}(1),x_{2}(2) \end{array} & \begin{array}{l} \cdots \\ \cdots \end{array} & \begin{array}{cc} \begin{array}{c} x_{1}(k)\\ x_{2}(k) \end{array} & \begin{array}{l} \cdots \\ \cdots \end{array} \end{array} & \begin{array}{l} x_{1}(n)\\ x_{2}(n) \end{array}\\ \begin{array}{c} \vdots \\ x_{i}(1),x_{i}(2) \end{array} & \begin{array}{c} \ddots \\ \cdots \end{array} & \begin{array}{cc} \begin{array}{c} \vdots \\ x_{i}(k) \end{array} & \begin{array}{c} \ddots \\ \cdots \end{array} \end{array} & \begin{array}{c} \vdots \\ x_{i}(n) \end{array}\\ \vdots & \ddots & \begin{array}{cc} \vdots & \ddots \end{array} & \vdots \\ x_{m}(1),x_{m}(2) & \cdots & \begin{array}{cc} x_{m}(k) & \cdots \end{array} & x_{m}(n) \end{array}\right]}_{m\times n}$$

The process of calculating the comprehensive risk value of food samples using GRA is as follows: 

(1) Dimensionless data. The difference in the physical significance of each indicator results in data that are not always of similar magnitude, which does not facilitate comparisons or makes it difficult to obtain correct conclusions when making comparisons. Dimensionless data processing is required for grey relational analysis. Equation (1) is used for dimensionless processing.

(1) $$y_{i}(k)=\frac{x_{i}(k)-\underset{1\leq k\leq n}{min}\{x_{i}(k)\}}{\underset{1\leq k\leq n}{max}\{x_{i}(k)\}-\underset{1\leq k\leq n}{min}\{x_{i}(k)\}}$$

where $y_{i}(k)$ is the dimensionless value of the ith indicator corresponding to the kth data element, i=1,2,…,m;k=1,2,…,n.

(2) The grey correlation coefficients of $y_{1}(k)$ and $y_{i}(k)$ at sample k are calculated as Equation (2).

(2) $$\xi_{i}(k)=\frac{\underset{i}{min}\;\underset{k}{min}\left|y_{1}(k)-y_{i}(k)\right|+\rho \underset{i}{max}\;\underset{k}{max}\left|y_{1}(k)-y_{i}(k)\right|}{\left|y_{1}(k)-y_{i}(k)\right|+\rho \underset{i}{max}\;\underset{k}{max}\left|y_{1}(k)-y_{i}(k)\right|}$$

where $\xi_{i}(k)$ is the grey correlation coefficient and ρ is called the adjustment parameter, which is used to adjust the difference between correlation coefficients (ρ∈(0,1)); the smaller the ρ, the greater the difference and the stronger the distinction, which is usually ρ=0.5.

(3) The correlation coefficient between the two sequences $y_{1}$ and $y_{i}$ is calculated as Equation (3):

(3) $$\gamma (y_{1},y_{i})=\frac{1}{n}\sum_{k=1}^{n}\xi_{i}(k)$$

(4) Each indicator acts as a reference sequence once, and the correlation coefficient matrix γ of all indicators can be obtained using Equation (3). In matrix γ, $\gamma_{iq}$ denotes the correlation between the ith and qth indicator.

$$\gamma ={\left[\begin{array}{cccc} \gamma_{11} & \begin{array}{cc} \cdots & \gamma_{1q} \end{array} & \cdots & \gamma_{1m}\\ \begin{array}{c} \vdots \\ \gamma_{i1} \end{array} & \begin{array}{c} \begin{array}{cc} \ddots & \vdots \end{array}\\ \begin{array}{cc} \cdots & \gamma_{iq} \end{array} \end{array} & \begin{array}{c} \ddots \\ \cdots \end{array} & \begin{array}{c} \vdots \\ \gamma_{im} \end{array}\\ \vdots & \begin{array}{cc} \ddots & \vdots \end{array} & \ddots & \vdots \\ \gamma_{m1} & \begin{array}{cc} \cdots & \gamma_{mq} \end{array} & \cdots & \gamma_{mm} \end{array}\right]}_{m\times m}$$

(5) Determining indicator weights. According to the correlation coefficient matrix γ, ${\bar{\gamma }}_{i}$ can reflect the weight of the ith indicator among all indicators, as calculated by Equation (4).

(4) $${\bar{\gamma }}_{i}=\frac{1}{m}\sum_{q=1}^{m}\gamma_{iq},(i=1,2,\ldots ,m)$$

Normalize ${\bar{\gamma }}_{i}$ by Equation (5) to obtain $W=[w_{1},w_{2},\ldots ,w_{m}]$ as the weight of each indicator.

(5) $$w_{i}=\sum_{q=1}^{m}\gamma_{iq}/\sum_{i=1}^{m}\sum_{q=1}^{m}\gamma_{iq}$$

(6) Calculation of risk values for food samples. The ratio of the detection value of the risk indicator to its limit value is used to express the risk of individual indicators on the sample, calculated by the equation $d_{ik}=x_{ik}/l_{i}$ (i=1,2,…,m;k=1,2,…,n), where $x_{ik}$ is the detection value of the kth sample corresponding to the ith indicator and $l_{i}$ is the maximum limit standard value of the ith indicator. Finally, the hazard level matrix for pollutants D obtained after the above calculation is multiplied by the indicator weight vector W to obtain the risk series of food samples, as shown in Equation (6).

(6) $$R=\left[r_{1},r_{2},\cdots ,r_{n}\right]=W\times D=\left[w_{1},w_{2},\cdots ,w_{m}\right]\left[\begin{array}{cccc} d_{11} & d_{12} & \cdots & d_{1n}\\ d_{21} & d_{22} & \cdots & c_{2n}\\ \begin{array}{l} \vdots \\ d_{i1}\\ \vdots \end{array} & \begin{array}{l} \vdots \\ d_{i2}\\ \vdots \end{array} & \begin{array}{l} \ddots \\ \cdots \\ \ddots \end{array} & \begin{array}{l} \vdots \\ d_{in}\\ \vdots \end{array}\\ d_{m1} & d_{m2} & \cdots & d_{mn} \end{array}\right]$$

where $R=[r_{1},r_{2},\ldots ,r_{n}]$ is the risk value matrix for n samples, D is the hazard level matrix for pollutants, and $W=[w_{1},w_{2},\ldots ,w_{m}]$ is the weight vector.

### 3.3. The TabNet-Based Food Safety Risk Prediction Model

**Figure 2:**
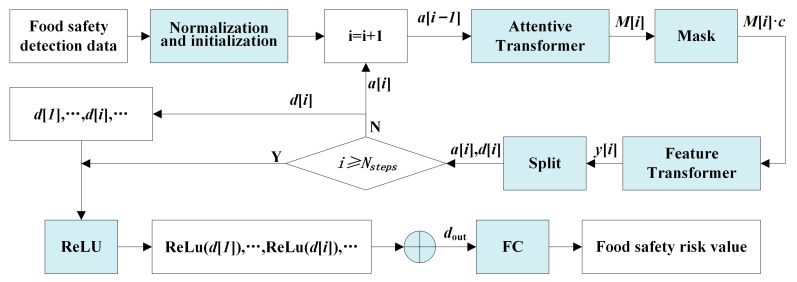
TabNet-based food safety risk prediction model.

TabNet is a novel high-performance standard deep learning architecture designed for tabular data; it has demonstrated remarkable performance in tasks such as the classification and regression of tabular-type data. TabNet is applied to predict food safety in this work. The architecture of the TabNet-based food safety risk prediction model is shown in Figure 2, which consists of $N_{steps}$ sequential decision steps, each consisting of the Attentive transformer (At) module, Mask module, Feature transformer (Ft) module, Split module, and ReLU activation function to realize, respectively, feature selection and feature processing. The normalization and initialization module contain two parts: the batch normalization (BN) layer and variable initialization. First, the sampling data were processed into a risk feature matrix through the BN layer, and the initialization of variables was performed. In addition, TabNet was encoded with the input of the ith step by the output of the (i−1)th step through the module to decide which feature was used, and the Mask-filtered features were then input into the Ft module for feature processing to obtain the processed risk features. When put into the Split module for division, the input of the next decision step a[i] and the output of the current decision step d[i] can be obtained in two parts. For the next At module for feature selection and the overall output to use, after $N_{steps}$ decision steps, the multi-step output vector d[i] is aggregated by the ReLU function to obtain the $d_{out}$, and then the Fully connected layer (FC) can be used for a transformation to take place. Finally the composite risk value of the sample was obtained. Specifically:

Step 1: Normalization and initialization.

The BN layer processes the sampling data matrix X into a risk feature matrix c as an input to each decision step, as shown in Equation (7).

(7) $$c=BN\left(X\right)$$

where $c\in {ℜ}^{B\times D}$, B denotes the batch size (number of food samples used at a time during training), and D denotes the number of dimensions of the features (risk indicators).

Initialize the related variables of TabNet: i=0, P[0]=1, a[0]=0.

Step 2: Let i=i+1, execute the decision step, and input a[i−1] into the At module to learn to obtain the Mask matrix M[i].

The Mask module implements the selection of significant features, which made the model focus on the risk indicators that mainly contribute to the risk when learning, thus improving the learning efficiency of the model. The importance of the features was realized by the At module, which implements feature selection for the current decision step by learning a Mask matrix as in Equation (8).

(8) $$M\left[i\right]=sparse\max\left(P\left[i-1\right]•hi\left(a\left[i-1\right]\right)\right)$$

where a[i−1] was the input information at the current decision step obtained by the Split module at (i−1)th step, and $h_{i}$ denoted a Fully connected (FC) layer and a BN layer operation, which served to achieve a linear combination of features so as to extract higher dimensional and abstracted features. $hi\left(a[i-1]\right)$ and P[i−1] were multiplied and then the desired M[i] was generated by the sparsemax algorithm. M[i] and feature elements were multiplied to achieve feature selection for the current decision step. P[i] denotes the use of features in the past decision step, which is updated by Equation (9).

(9) $$P[i]=\prod_{j=1}^{i}\left(\gamma -M[j]\right)$$

where γ is a relaxation parameter. When γ=1, a feature is forced to be used in only one decision step. In addition, the sparsemax algorithm was employed to assign weights to individual features of each sample, ensuring that the total sum of weights for all features in a sample equaled 1, i.e., $P[i]\sum_{j=1}^{D}M{\left[i\right]}_{b,j}=1$, where D denotes the dimensionality of the features, thus realizing instance-wise feature selection.

Step 3: The risk feature matrix c and $M\left[i\right]$ were passed through the Mask module to select the significant features of food safety risks.

The feature selection for the current decision step was achieved by multiplying $M\left[i\right]$ and the risk feature matrix c to obtain the food safety risk significant feature $M\left[i\right]•c$.

Step 4: The significant feature $M\left[i\right]•c$ was input into the Ft module for processing to obtain the risk feature $y\left[i\right]$ as in Equation (10):

(10) $$y[i]=f_{i}(M[i]\cdot c)$$

where $f_{i}$ is the Ft module operation and c is the risk feature matrix.

Step 5: The processed risk features $y\left[i\right]$ were put into the Split module for segmentation.

The processed risk features $y\left[i\right]$ were divided into two parts by the Split module; one part was used for the output of the current decision step $\left(d[i]\right)$ and the other part was used as the input information for the next decision step $\left(a[i]\right)$, where $d[i]\in {ℜ}^{B\times Nd}$, $a[i]\in {ℜ}^{B\times Na}$, Nd is the number of features in the total decision output, and Na is the number of features input to the At module for the next step.

Step 6: Determine whether i is less than $N_{steps}$, then go to Step 2 to perform the next decision step, or otherwise go to Step 7.

Step 7: TabNet draws on the idea of tree model aggregation to aggregate the output vectors d[i] of all the decision steps into $d_{out}$, where dout=∑i=1NstepsReLU(d[i]), and then finally a FC layer is mapped to the final output, which was the predicted fused risk value.

After the above steps, a food safety risk prediction model based on TabNet was constructed, with the detection data matrix X as the input, and the comprehensive risk vector R as the expected output. The relevant hyperparameters of TabNet were set and adjusted to train the risk prediction model.

## 4. Case Study and Model Evaluation

This work presents an analysis using the safety detection data of cooked meat products provided by the food detection department of a Chinese province in 2018 and 2019. Firstly, the raw data were processed, adhering to the principles of the comprehensiveness, scientific state, and operability of the risk evaluation indicator system [18]. Moreover, nine risk indicators were selected for food additives (nitrite, sorbic acid, and benzoic acid), heavy metal elements (lead, cadmium, chromium, and total arsenic), and microorganism (coliform and total bacterial count) categories, which have an important impact on the risk of meat products. Secondly, the GRA method was employed to calculate the weights of each indicator, and the risk value of meat samples was obtained by fusing the results with the weights; then, the food safety risk prediction model was constructed based on TabNet, the detection content data of each hazard in the meat product sample were used as the input of the model, and the comprehensive risk value calculated via the GRA was used as the expected output to perform the model training process.

### 4.1. Data Preprocessing

The detection data used in this work comprised 87,260 raw data records, a part of the raw data is shown in Table 1. The data included over 50 attributes (e.g., sample number, sampling time, product name, detection items, detection results, etc.) and contained detection items other than the nine indicators to be used in this experiment. Each detection item possessed a unique discrete domain, while the format of the detection result data primarily utilized in model construction lacked standardization and contained many superfluous attributes, redundant data, etc. Therefore, preprocessing the detection results is essential prior to modeling.

To address the issues with the data, the following processing steps were undertaken: (1) Useless attributes were eliminated, retaining only twelve relevant attributes, including nine risk indicators, sample number, limit standards, and standard detection limits. (2) Data formats were converted, and the redundant non-numerical symbols in the data were removed, e.g., “<0.005” to “0.005”. (3) Redundant data were removed, for example, if there were multiple numbers in the detection result; the maximum value was taken as its detection result. (4) “Undetected” results were addressed by filling them with half of the standard detection limit rather than assigning a value of zero. (5) Outlier detection was performed on the data to identify and exclude abnormal samples. The processed data are presented in Table 2, consisting of a total of 7933 samples. Among these samples, 7885 samples from 2018 to November 2019 were utilized for model construction (7835 for model training and 50 for model test), while 48 samples were retained from December 2019 to be applied in the risk early warning and visualization system.

### 4.2. Calculating the Comprehensive Risk Value

**Figure 3:**
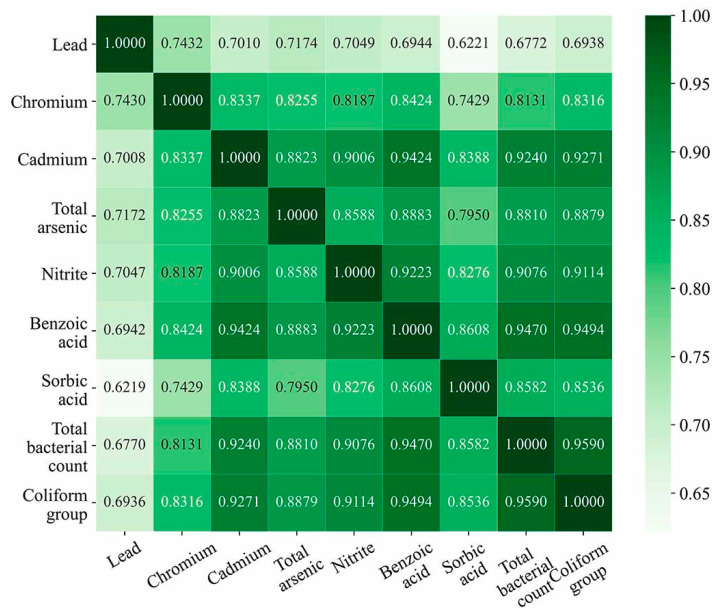
Matrix heat map showing the correlation between risk indicators.

Based on the processed cooked meat products detection data, correlation analysis was performed using the GRA method on 7885 sample data from January 2018 to November 2019 to obtain the weights of nine evaluation indicators, and the comprehensive risk values of the samples were obtained by fusing the data on the detected contents of hazards in cooked meat product samples with the indicator weights. The correlation coefficient between each risk indicator was obtained using Equations (1)–(3), and the correlation coefficient represented the correlation degree between the indicators; the larger the correlation coefficient, the greater the correlation degree between the two indicators. The correlation degree and heat map matrix between the evaluation indicators are shown in Figure 3. Then, based on the correlation coefficient matrix, the weights of each risk evaluation indicator were calculated using Equations (4) and (5), and the results are shown in Table 3. Finally, the risk value of each food sample was calculated using Equation (6) as the expected output of the model. The results of the risk assessment are shown in Table 4.

### 4.3. Model Construction and Evaluation

In constructing the food safety risk prediction model using the pytorch-tabnet package, 7835 samples were used as the training set for TabNet model training, and the remaining 50 samples were used as the test set; Table 5 shows the parameter settings of the risk prediction model this study proposed. To verify the predictive power of this model, comparison experiments were performed between the TabNet-based model and six typical predictive models on the same detection dataset. The comparison models included three tree models, RF, GBDT, and XGBoost, and three neural networks: BP, ELM, and RBF. Among them, RF is an integrated learning model with a decision tree as the base learner, which integrates the results of multiple decision trees to obtain the final training results; the GBDT model mainly achieves the purpose of data learning by the linear combination of base learners and continuously reducing the residuals generated by the training process. XGBoost has more efficient and accurate prediction capabilities by adding a regular term to the loss function as well as supporting parallel computation; BP is a multi-layer feedforward neural network trained according to the error back propagation algorithm, which is one of the more widely used neural network models; and ELM and RBF are kinds of single hidden layer feedforward neural networks—ELM has the advantages of few training parameters and fast learning speed and RBF uses radial basis function as the activation function of hidden layer neurons, which is a kind of local approximation network with a strong generalization ability.

**Figure 4:**
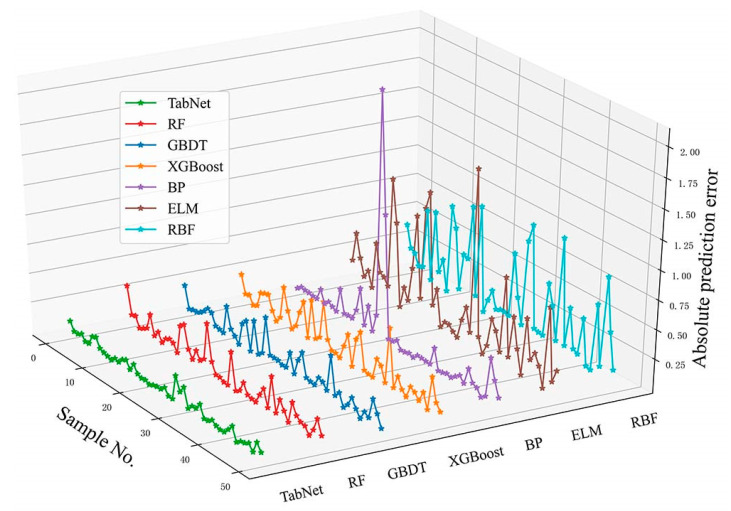
Absolute prediction error curves of the seven models.

**Figure 5:**
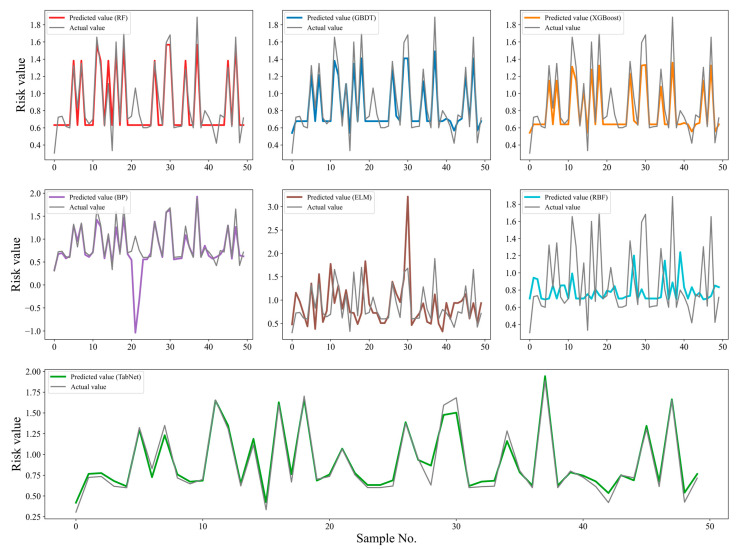
Comparison of the fitting ability (actual value vs. predicted value) among the seven models.

The root mean squared error (RMSE) and mean absolute error (MSE) are used to judge the performance of each model on the test set, and the smaller the values, the better the risk prediction ability of the model, which are calculated by Equations (11) and (12), respectively. The experimental results are shown in Table 6. The RMSE value and MAE value of the TabNet-based model are the smallest among the seven models, which are 0.0710 and 0.0532, respectively, indicating that TabNet can predict the risk value of meat samples more accurately. The risk prediction error curves of the seven models are shown in Figure 4. The error is obtained by subtracting the true value from the model prediction and taking the absolute value, and it can be seen that the error curve of TabNet fluctuates the least. Meanwhile, from Figure 5, it can be found that the curves of the predicted values of TabNet almost overlap with the curves of the true values, while the curves of the predicted values of the other models have a larger gap with the curves of the true values, indicating that its fitting ability is stronger than that of the other comparison models. Therefore, it can be concluded that the TabNet-based food safety risk prediction model is better than the other comparison models in terms of risk prediction accuracy, stability, and generalization ability.

(11) $$RMSE=\sqrt{\frac{1}{n}\sum_{i=1}^{n}{\left({\hat{y}}_{i}-y_{i}\right)}^{2}}$$

(12) $$MAE=\frac{1}{n}\sum_{i=1}^{n}\left|{\hat{y}}_{i}-y_{i}\right|$$

where n is the number of samples, $y_{i}$ is the true risk value of the $i^{th}$ sample, and ${\hat{y}}_{i}$ is the predicted risk value of the $i^{th}$ sample.

## 5. FSRvis System

**Figure 6:**
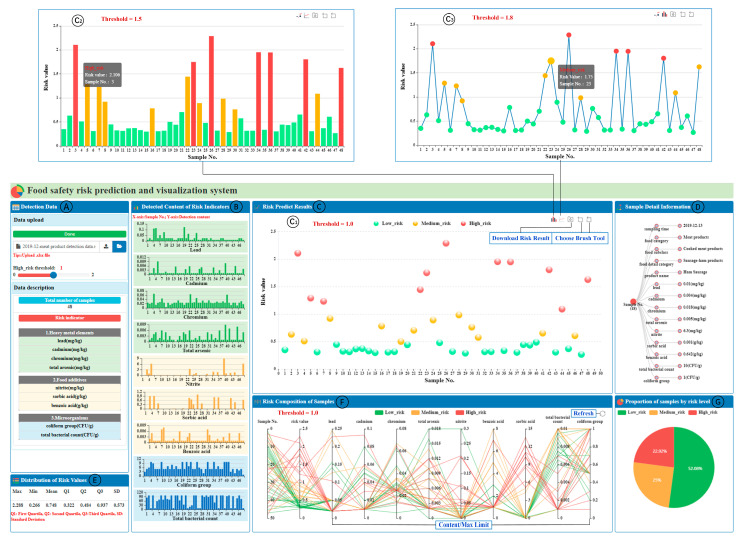
Multiple views in the interface of the FSRvis system. (**A**) Detection data; (**B**) detected content of risk indicators; (**C**) risk predict results; (**D**) sample detail information; (**E**) distribution of risk values; (**F**) risk composition of samples; (**G**) portion of samples by risk level.

The framework of the FSRvis system developed in this work is shown in Figure 1B. It comprised several views, including the detection data view; risk indicator detection content view; risk prediction results view; sample details view; risk value distribution view; sample risk composition view; and the portion view of samples for each risk level, which can realize the prediction and visual analysis of food safety risk based on sampling data. Figure 6 shows the interface of the system obtained by uploading the detection results of 48 samples of cooked meat products in December 2019. Due to space limitations, please refer to the Supplementary Material for a detailed description of the interaction of each view of the system.

In the detection data view (Figure 6A) in the FSRvis system, analysts uploading food detection results in the tabular form receive the content presented in the other views, and sliders are used to adjust the warning thresholds. The uploaded data sample size as well as the risk indicators are also shown to facilitate a better understanding of the content of the other views. The detection content of the risk indicator view (Figure 6B) presents the detection content of nine risk indicators in the form of a bar chart, which allows you to clearly see the distribution of the detection content of each evaluation indicator, as well as the comparison of the content of each sample in the same indicator.

The risk prediction results view (Figure 6C) presents the risk prediction results by the risk prediction model for the uploaded samples, with the horizontal axis indicating the sample number and the vertical axis indicating the predicted risk value. The prediction results are visualized using three markers, point, line, and surface, respectively, and two visual channels, position and color, to present the prediction results. According to the risk calculation in this work, when the sample risk is greater than one, it means that there is at least one risk indicator in the sample with a detection content greater than the maximum limit, so the risk level is coded by color in the three sub-views: red denotes that the risk value is greater than the warning threshold (high risk), yellow denotes that the sample risk value is between one-half of the warning threshold and the warning threshold (medium risk), and green denotes that the sample risk is less than one-half of the warning threshold (low risk), and the analyst can slide the slider in view (Figure 6A) to adjust the warning threshold according to the actual situation. In this way, the high-risk food samples (red-marked samples in view (Figure 6C)) can be found visually and effectively. The subview (Figure 6C_1_) can effectively discover the distribution of different sample risks in the form of a scatter plot; the subview (Figure 6C_2_) can visually compare the risk level of adjacent or different samples in the form of a bar chart; and the subview (Figure 6C_3_) can obviously observe the trend of sample risks in the form of a line graph. For problems such as graphical overlap caused by a large number of samples, a brush tool is designed to enable the swiping of samples, which can increase the distance between markers in the view, and this tool is also applicable to views (Figure 6C_2_) and (Figure 6C_3_). For further analysis, a function for downloading risk results was designed in each subview of view (Figure 6C).

The sample details information view (Figure 6D) uses a hierarchical tree to explore the details of a single sample. By selecting a sample in view (Figure 6C) by clicking on it, you can display information about the detection time, food category, food name, and sampling results of each risk indicator for that sample in view (Figure 6D). The root node in the tree diagram denotes the sample, the color is synchronized with the view (Figure 6C), the second layer denotes the attribute name, and the third layer denotes the attribute value. The distribution information of the predicted risk results for the data samples is presented in the distribution of the risk values view (Figure 6E), through which the overall situation of the sample risk can be understood, e.g., the third quartile is 0.937, indicating that three-quarters of the sample risk is below 0.937.

In the risk composition of the samples view (Figure 6F), the relative risk of each indicator is presented using parallel coordinates, which is the hazard level matrix for pollutants D in Equation (6), where each vertical axis represents an indicator and each line through each indicator represents a sample, which passes through each axis, from which the impact of each indicator on the risk of the sample can be analyzed. As in view (Figure 6C), here the color channels are used to indicate the level of risk. The proportion of the samples by the risk-level view (Figure 6G) mainly presents the percentage of samples with different risk levels obtained from the uploaded data samples, as predicted by the TabNet-based model.

## 6. Discussion

First, the proposed GRA-based quantitative risk assessment method can calculate fine-grained risk values of food products in detection data, enabling a more accurate identification of food safety risks. This method calculated the correlation between each risk indicator by using the GRA approach and obtained the weight of each indicator, subsequently calculating the risk value based on the weighted sum of all indicator detections. In comparison with qualitative risk assessment methods, this quantitative approach allowed for a more precise evaluation of food risks, overcoming the limitations of subjectivity and difficulties in quantifying risks to a certain extent.

Second, the proposed food safety risk prediction method, TabNet-GRA, provided a rapid and direct comprehensive risk value for food based on detection data, exhibiting a superior predictive ability when compared with typical prediction models. The TabNet-GRA method first derives the comprehensive risk value of fused detection data using GRA. It then constructs a risk prediction model based on TabNet, trained using the detection data as the input and the comprehensive risk value calculated via the GRA as the expected output. Subsequently, users can employ this trained model to directly predict the comprehensive risk value of food based on new detection data, eliminating the need for previous complex calculation processes. As a result, food safety risks can be promptly identified. Comparative experimental results demonstrated that the TabNet-based prediction model exhibits lower error rates than the current typical models, including RF, GBDT, XGBoost, BP, ELM, and RBF, showcasing its superior fitting ability and ability to predict food safety risk more accurately and efficiently.

Third, the developed FSRvis system offers support for food safety risk assessment, prediction, and visual analysis in a more intuitive and effective manner. The system integrated the risk prediction model constructed based on the TabNet-GRA method and employed multi-view collaborations, providing multiple views of detection data, detection content for each risk indicator, risk prediction results, risk composition, sample detail information, etc. This approach enabled risk prediction and multi-faceted interactive visual analysis of new detection data, thereby enhancing the efficiency and accuracy of risk analysis. Food safety supervision departments can utilize this system to conduct in-depth analysis of detection data and subsequently implement targeted food safety monitoring, early warning, and control measures based on risk analysis outcomes. For example, the FSRvis system can be employed to focus on monitoring and controlling high-risk food products and safety hazards, ultimately improving the cost-efficiency of supervision efforts.

## 7. Conclusions and Future Work

In conclusion, this work addressed the critical aspect of data-driven food safety risk early warning, which was a pivotal method for ensuring effective food safety supervision. This study proposed an innovative risk prediction method for food safety, namely TabNet-GRA, which harnessed the advantages of TabNet and GRA to empower accurate and expeditious fine-grained risk prediction based on detection data. To substantiate its efficacy, this study conducted a comprehensive case study and method evaluation using a dataset comprising 87,260 original records of cooked meat products detection from a Chinese province. The comparative evaluation unequivocally demonstrated the superiority of the TabNet-based prediction model over six typical models (RF, GBDT, XGBoost, BP, ELM, and RBF) under equivalent conditions.

Additionally, this study has implemented an intelligent visualization system named the FSRvis system which is built upon the TabNet-GRA method. This advanced system facilitated food safety risk prediction and multi-dimensional interactive visual analysis, elevating the efficiency and scope of food safety risk analysis to new heights.

In future work, the intention is to explore two pivotal research directions. Firstly, the goal involves parameter optimization of the risk prediction model by employing particle swarm optimization (PSO) and Bayesian optimization techniques, aiming to further elevate the prediction accuracy. Secondly, attention will be directed towards devising an automated warning report generation method, which is capable of generating comprehensive analysis reports for users following risk analysis and prediction of detection data. These improvements will substantially amplify the practicality and real-world applicability of the TabNet-GRA method.

## Figures and Tables

**Table 1:** Raw detection data (partial).

No.	Sample No.	Sampling Time	Product Name	Detection Item	Detection Result	Maximum Limit	Standard Detection Limit	Unit
1	1	3 January 2018	Duck in sauce	lead	0.0425	0.5	0.05	mg/kg
2	2	3 January 2018	Beef Jerky	chromium	0.3570	1.0	0.03	mg/kg
3	3	3 January 2018	Ham Sausage	nitrite	4.2	30	0.2	mg/kg
4	3	3 January 2018	Ham Sausage	sorbic acid	0.86	0.075	0.01	g/kg
5	4	31 January 2018	Bacon	benzoic acid	<0.005	shall not be used	0.005	g/kg
6	4	31 January 2018	Bacon	cadmium	<0.008	0.1	0.003	mg/kg
7	5	4 February 2018	Roasted leg with sauce	total bacterial count	80; 70; 90; 50; 180	10,000	/	CFU/g
8	5	4 February 2018	Roasted leg with sauce	total arsenic	Not Detected	0.5	0.04	mg/kg
9	5	4 February 2018	Roasted leg with sauce	coliform group	<10	10	/	CFU/g

**Table 2:** Processed detection data (partial).

Sample No.	Lead	Cadmium	Chromium	Total Arsenic	Nitrite	Benzoic Acid	Sorbic Acid	Total Bacterial Count	Coliform Group
1	0.0425	0.0025	0.0522	0.0010	9.600	0.0050	0.0050	100	5
2	0.1270	0.0090	0.3570	0.0450	0.001	0.0025	0.0050	85	10
3	0.0744	0.0057	0.1400	0.0640	4.200	0.0100	0.8600	10	0
4	0.0806	0.0080	0.4180	0.0875	3.100	0.0050	0.0050	100	5
5	0.0332	0.0015	0.0250	0.0200	0.100	0.0025	0.0050	180	10
6	0.2440	0.0115	0.7680	0.0111	6.100	0.0050	0.0050	100	5
7	0.1720	0.0015	0.0580	0.0010	20.00	0.0025	0.0405	10	0
8	0.0500	0.0050	0.2000	0.0400	4.160	0.0100	0.0050	70	10
9	0.0611	0.0015	0.0250	0.0370	0.100	0.0025	0.0232	200	10
10	0.1300	0.0094	0.0990	0.0010	5.400	0.1000	0.0050	100	5

**Table 3:** The weight of each risk indicator.

Indicator	Lead	Cadmium	Chromium	Total Arsenic	Nitrite	Benzoic Acid	Sorbic Acid	Total Bacterial Count	Coliform Group
Weight	0.0950	0.1153	0.1080	0.1122	0.1138	0.1167	0.1073	0.1155	0.1162

**Table 4:** Risk assessment results (partial).

Sample No.	1	2	3	4	5	6	…	7885
Risk value	0.6131	0.7433	1.5476	0.3113	0.6040	1.3979	…	0.5988

**Table 5:** Parameter value setting of food safety risk prediction model constructed based on the TabNet-GRA method.

Parameter	Description	Value
N_d	Width of the decision prediction layer	8
N_a	Width of the attention embedding for each mask	8
N_steps	Number of steps in the architecture	3
Lr	Learning rate	0.01
Max_epochs	Maximum number of epochs for training	1000
Batch_size	Number of examples per batch	7835
Virtual_batch_size	Size of the mini batches used for “GBN”	128
Optimizer_fn	Pytorch optimizer function	Adam

**Table 6:** Risk prediction error of the seven models.

	RF	GBDT	XGBoost	BP	ELM	RBF	TabNet
RMSE	0.1435	0.1485	0.1842	0.3532	0.4533	0.4362	**0.0710**
MAE	0.1038	0.1147	0.1376	0.1385	0.3088	0.3217	**0.0532**

## Data Availability

Data are contained within the article.

## Supplementary Materials

The following supporting information can be downloaded at: https://www.mdpi.com/article/10.3390/foods12163113/s1, Food safety risk prediction and visualization system decomposition diagram and description.

## References

[1] Fukuda K. (2015). Food safety in a globalized world. Bull. World Health Organ..

[2] Wang X., Bouzembrak Y., Lansink A.O., van der Fels-Klerx H.J. (2022). Application of machine learning to the monitoring and prediction of food safety: A review. Compr. Rev. Food. Sci. Saf..

[3] World Health Organization (WHO) Food Safety. https://www.who.int/health-topics/food-safety.

[4] Liu Z., Meng L.Y., Zhao W., Yu F.Q. (2010). Application of ANN in food safety early warning. Proceedings of the 2010 2nd International Conference on Future Computer and Communication.

[5] Marvin H.J.P., Janssen E.M., Bouzembrak Y., Hendriksen P.J.M., Staats M. (2017). Big data in food safety: An overview. Crit. Rev. Food Sci. Nutr..

[6] Nogales A., Díaz-Morón R., García-Tejedor Á.J. (2022). A comparison of neural and non-neural machine learning models for food safety risk prediction with European Union RASFF data. Food Control.

[7] Bouzembrak Y., Marvin H.J.P. (2019). Impact of drivers of change, including climatic factors, on the occurrence of chemical food safety hazards in fruits and vegetables: A Bayesian Network approach. Food Control.

[8] Ru G., Crescio M.I., Ingravalle F., Maurella C. (2017). Machine Learning Techniques applied in risk assessment related to food safety. EFSA Support. Publ..

[9] Liu Y.H., Qu Y., Jiang J.M., Zong W.L., Zhu X.J. (2021). Prediction of unqualified index of food inspection based on optimized random forest algorithm. J. Food Saf. Qual..

[10] Gao Y.N., Wang W.Q., Wang J.X. (2021). A Food Safety Risk Prewarning Model Using LightGBM Integrated with Fuzzy Hierarchy Partition: A Case Study for Meat Products. Food Sci..

[11] Wang X.Y., Wang Z.Y., Zhao Z.Y., Zhang X., Chen Q., Li F. (2022). A food safety risk forecast model integrated with improved AHP and XGBoost algorithm: A case study of rice. J. Food Sci. Technol..

[12] Geng Z.Q., Zhao S.S., Tao G.C., Han Y.M. (2017). Early warning modeling and analysis based on analytic hierarchy process integrated extreme learning machine (AHP-ELM): Application to food safety. Food Control.

[13] Geng Z.Q., Liu F.F., Shang D.R., Han Y.M., Shang Y., Chu C. (2021). Early warning and control of food safety risk using an improved AHC-RBF neural network integrating AHP-EW. J. Food Eng..

[14] Geng Z.Q., Shang D.R., Han Y.M., Zhong Y.H. (2018). Early warning modeling and analysis based on a deep radial basis function neural network integrating an analytic hierarchy process: A case study for food safety. Food Control.

[15] Niu B., Zhang H., Zhou G.Y., Zhang S.W., Yang Y.F., Deng X.J., Chen Q. (2021). Safety risk assessment and early warning of chemical contamination in vegetable oil. Food Control.

[16] Xie T.T., Yu H., Wilamowski B. Comparison between traditional neural networks and radial basis function networks. Proceedings of the 2011 IEEE International Symposium on Industrial Electronics (ISIE 2011).

[17] Arik S.Ö., Pfister T. (2021). TabNet: Attentive Interpretable Tabular Learning. AAAI Conf. Artif. Intell..

[18] Khalili E., Ramazi S., Ghanati F., Kouchaki S. (2022). Predicting protein phosphorylation sites in soybean using interpretable deep tabular learning network. Brief. Bioinform..

[19] Yan J.Z., Xu T.Y., Yu Y.C., Xu H.X. (2021). Rainfall Forecast Model Based on the TabNet Model. Water.

[20] Wang W., Wu P., Zhao X. (2013). Soil infiltration based on BP neural network and grey relational analysis. Rev. Bras. De Ciência Do Solo.

[21] Liu S., Cai H., Yang Y., Cao Y. (2013). Research progress of grey relational analysis model. Syst. Eng. Theory Pract..

[22] Han Y.M., Cui S.Y., Geng Z.Q., Chu C., Chen K., Wang Y.J. (2019). Food quality and safety risk assessment using a novel HMM method based on GRA. Food Control.

[23] Lin X.Y., Cui S.Y., Han Y.M., Geng Z.Q., Zhong Y.H. (2018). An improved ISM method based on GRA for hierarchical analyzing the influencing factors of food safety. Food Control.

[24] Liu M.C., Shi J.X., Li Z., Li C.X., Zhu J., Liu S.X. (2017). Towards Better Analysis of Deep Convolutional Neural Networks. IEEE Trans. Vis. Comput. Graph..

[25] Yuan J., Chen C.J., Yang W.K., Liu M.C., Xia J.Z., Liu S.X. (2021). A survey of visual analytics techniques for machine learning. Comput. Vis. Media.

[26] Chen Y., Zhang Q.H., Guan Z.L., Zhao Y., Chen W. (2022). GEMvis: A visual analysis method for the comparison and refinement of graph embedding models. Vis. Comput..

[27] Wu C.X., Chen Y., Dong Y., Zhou F.F., Zhao Y., Liang C.J. (2023). VizOPTICS: Getting insights into OPTICS via interactive visual analysis. Comput. Electr. Eng..

[28] Chen Y., Dou H., Chang Q., Fan C. (2022). PRIAS: An Intelligent Analysis System for Pesticide Residue Detection Data and Its Application in Food Safety Supervision. Foods.

[29] Chen Y., Lv C., Li Y., Chen W., MA K.L. (2020). Ordered matrix representation supporting the visual analysis of associated data. Sci. China Inf. Sci..

[30] Luo Z., Chen Y., Li H., Li Y., Guo Y. (2022). TreeMerge: A Visual Comparative Analysis Method for Food Classification Tree in Pesticide Residue Maximum Limit Standards. Agronomy.

[31] Chen Y., Guo Y., Fan Q., Zhang Q., Dong Y. (2023). Health-Aware Food Recommendation Based on Knowledge Graph and Multi-Task Learning. Foods.

[32] Chen Y., Dong Y., Sun Y., Liang J. (2018). A Multi-comparable visual analytic approach for complex hierarchical data. J. Vis. Lang. Comput..

