# Supplementary material for: Prediction and Visual Analysis of Food Safety Risk Based on TabNet-GRA
Source: Foods. 2023 Aug 18;12(16):3113. doi: 10.3390/foods12163113 (PMC10453234; doi:10.3390/foods12163113)
Supplement: Supplementary file 1 [file foods-12-03113-s001.zip › foods-2499227-supplementary.pdf]

Supplementary Material

We add further detailed descriptions of the interaction operations of each view in the FSRvis system proposed in the paper here. Among them, Figure S1 shows the multi-view interface of the system, and Figure S2 - Figure S14 is mainly a detailed description of the interaction of each view and the specific presentation of the view after the interaction. The details are as follows.

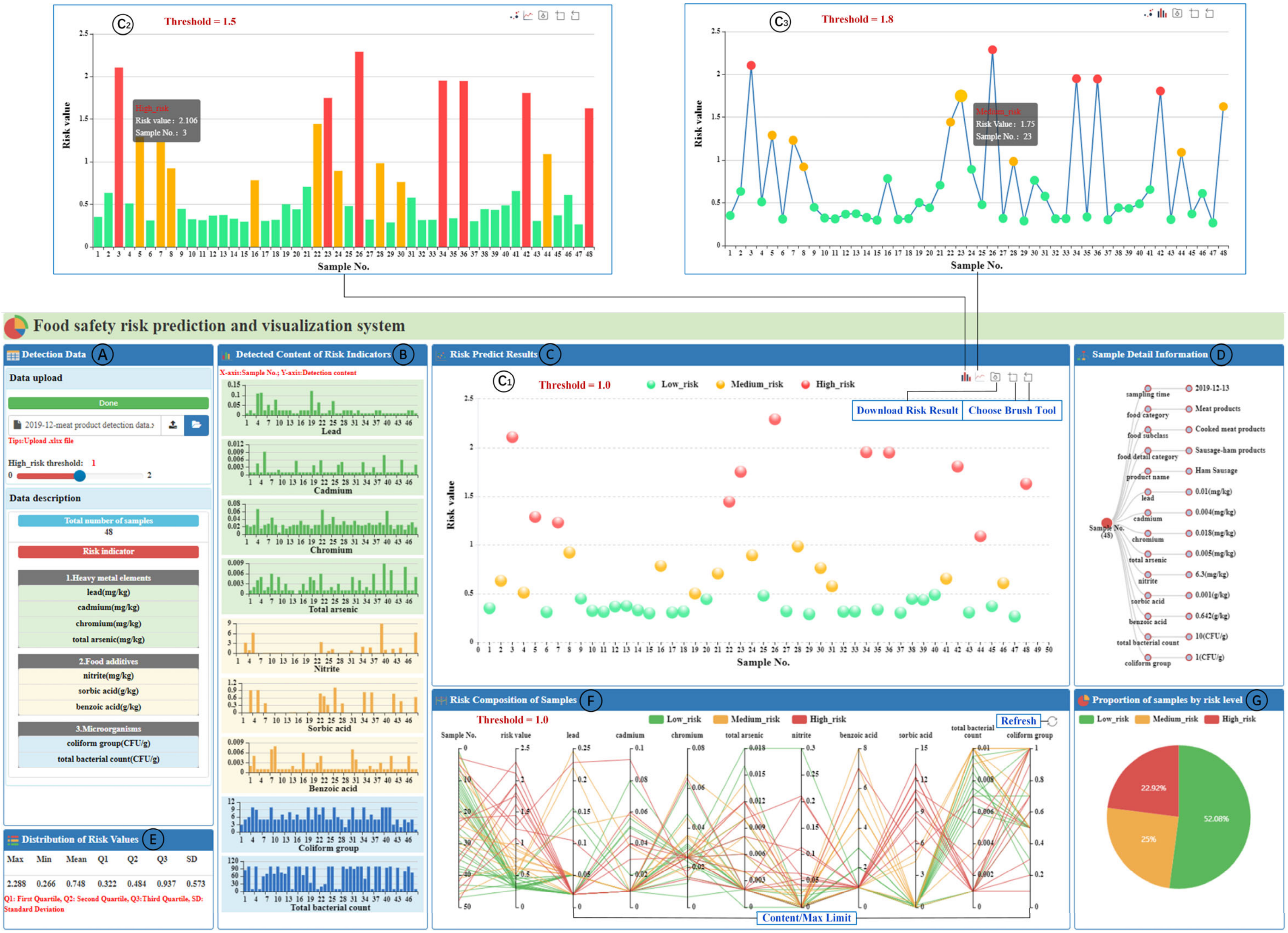

Figure S1. Multiple views in the interface of FSRvis system. (A) Detection Data; (B) Detected content of risk indicators; (C) Risk predict results; (D) Sample detail information; (E) Distribution of risk values; (F) Risk composition of samples; (G) Proportion of samples by risk level.

1. The detection data view A includes two parts: data upload and data description, upload the food sampling results, and you can get the content presented in other views, sliders are used to adjust the high-risk threshold, and views C, F, and G will change according to the threshold; the data description section shows the number of uploaded data samples and the risk indicator categories and names. The risk indicator detection content view B presents the detection content of all risk indicators, with the X-axis indicating the sample number and the Y-axis indicating the detection content. The risk value distribution view E presents information on the distribution of the predicted risk results for the data samples, which provides an overall picture of the risk of the samples. As shown in Figure S2.

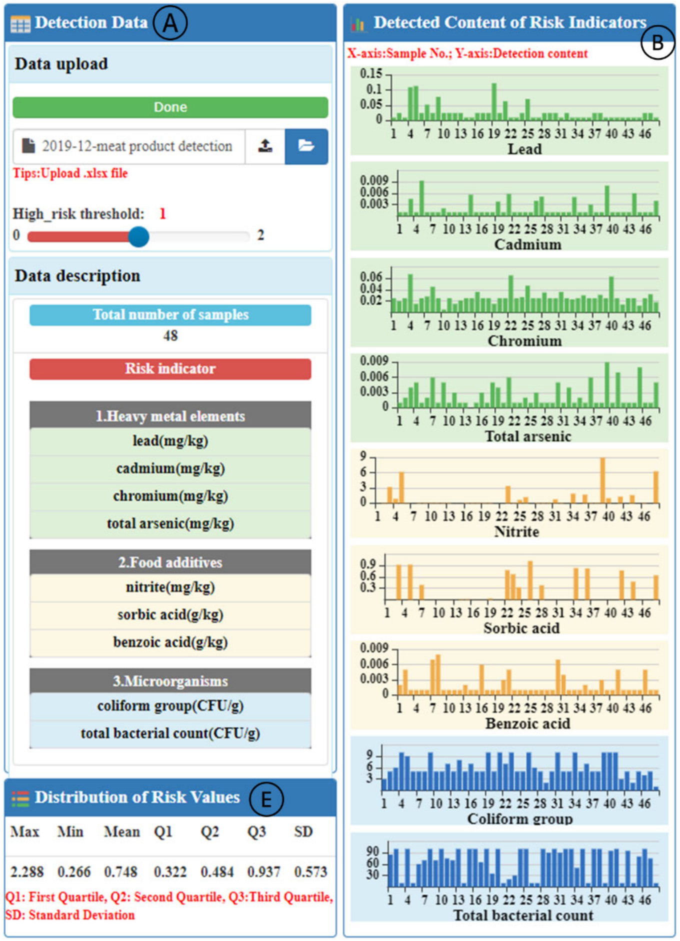

Figure S2. View A, View B, and View E in Figure S1.

2. The risk prediction results of the food safety risk prediction model for the uploaded samples are presented in view C. The horizontal axis indicates the sample number and the vertical axis indicates the predicted risk value, and contains three sub-views (scatter, bar, and line graph visualizations), which are switched by the logo in the upper right corner of view C. The risk level is coded by color in the three sub-views, and the risk level, risk value, and sample number are indicated when the mouse is moved to a sample, and view C changes (in color) when the slider with the high-risk threshold in view A is changed, as shown in Figure S3, S4 and S5. For problems such as graphic overlap caused by a large number of samples, a choose brush tool was designed to increase the distance between markers in the view, as shown in Figure S6. For further analysis, the function of downloading risk results is designed in each sub-view of view (C).

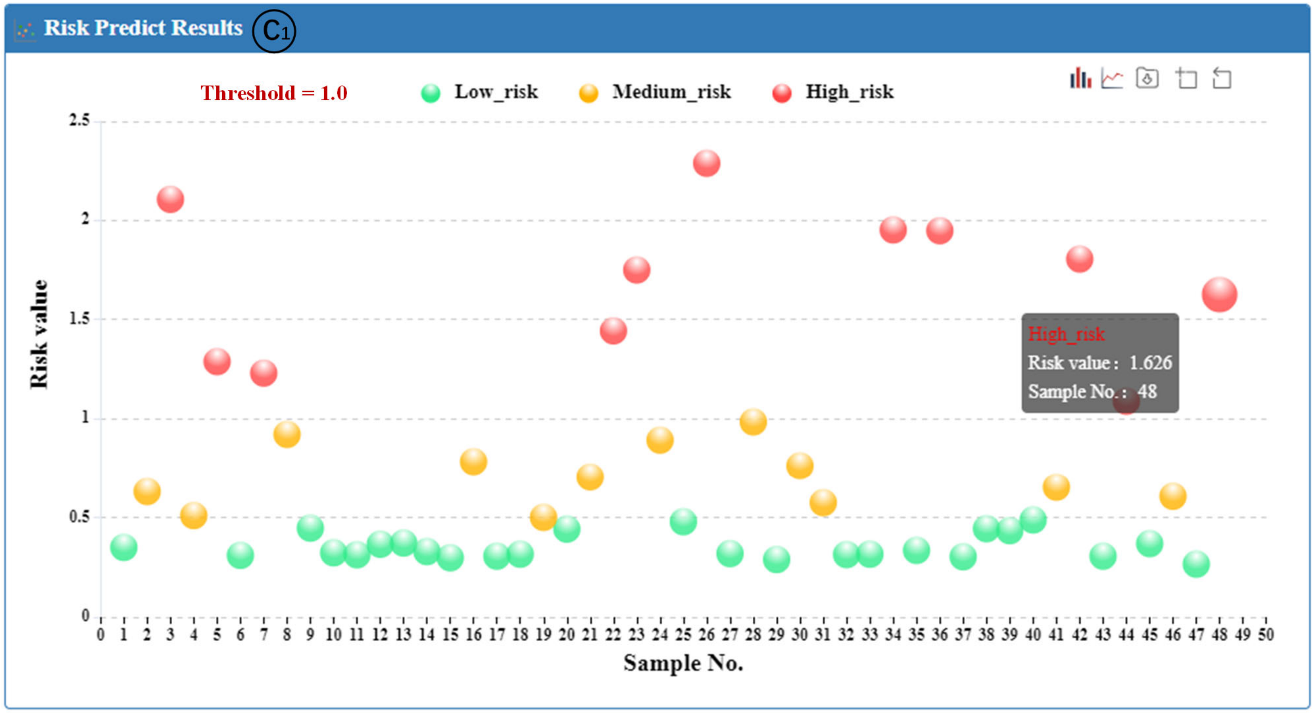

Figure S3. View C_1 in Figure S1, which shows risk prediction results displayed with the scatter plot when the threshold is 1.0.

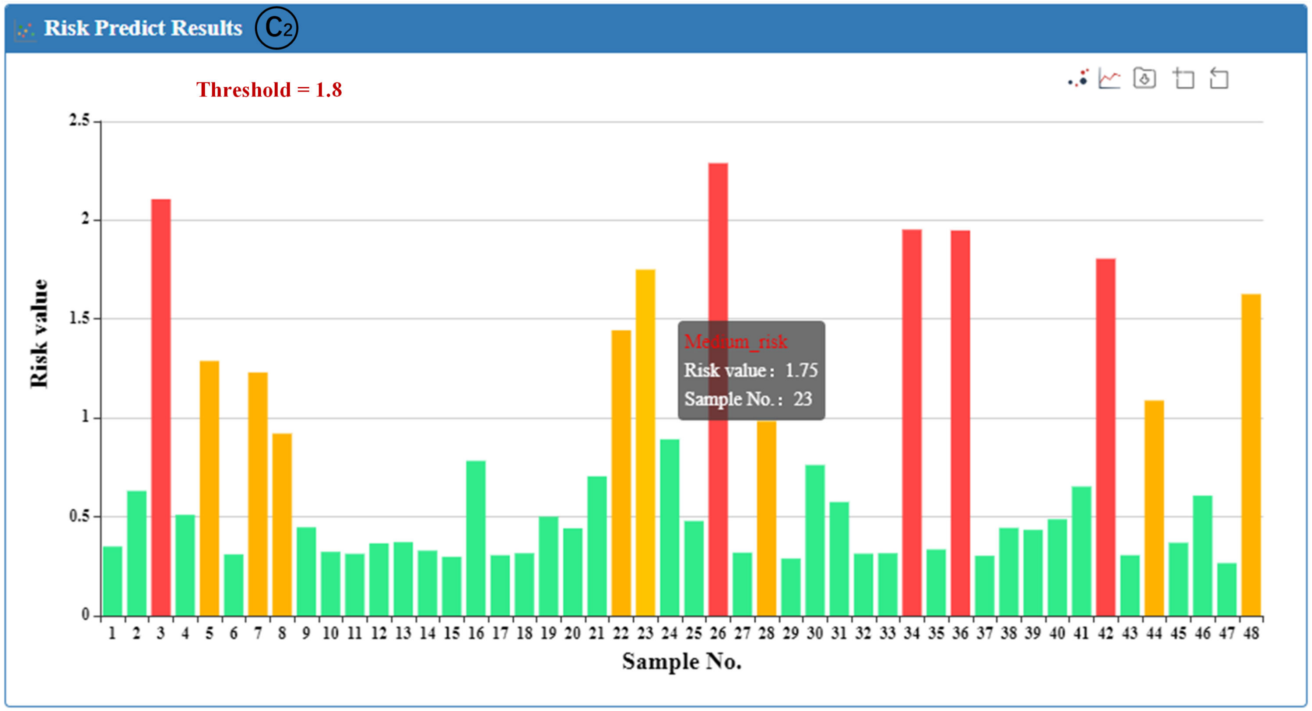

Figure S4: View C_2 in Figure S1, which shows risk prediction results displayed with the bar chart when the threshold is 1.8

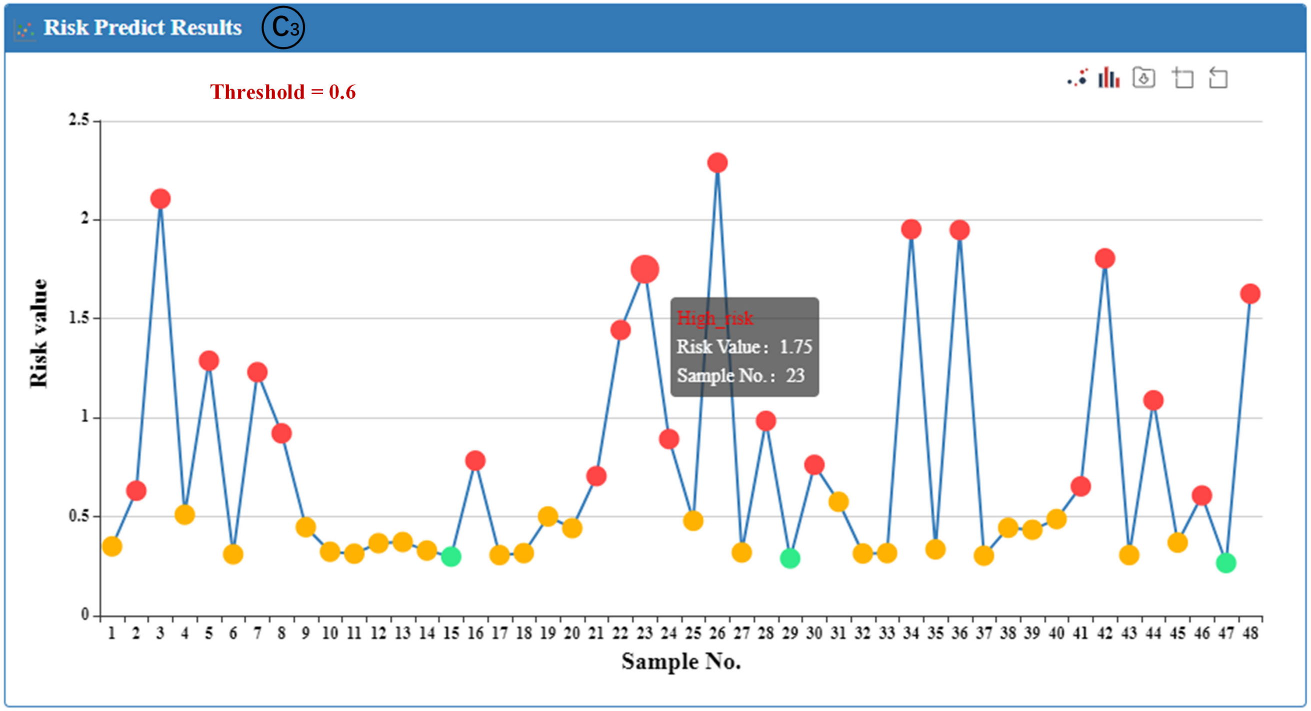

Figure S5. View C3 in Figure S1, which shows risk prediction results displayed with the line graph when the threshold is 0.6.

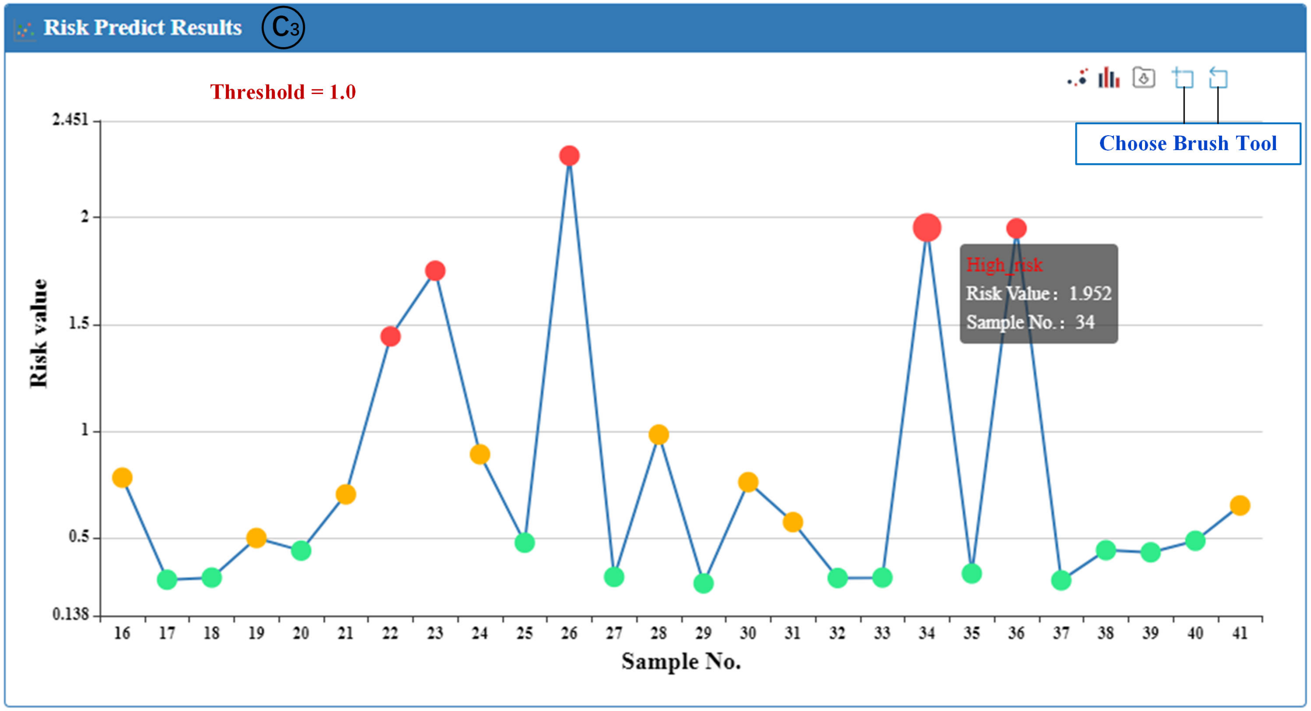

Figure S6: Risk prediction results shown in View C_3 after choosing the brush tool

3. Sample details information view D uses a hierarchical tree to display the details of individual samples. By clicking on the sample in view C, you can display the sample in view D of the detection time, food category, food name, the sampling results of each risk indicator, and other information. The root node in the tree diagram indicates the sample, and the color is synchronized with view C. The second layer indicates the attribute name, and the third layer indicates the attribute value. Figure S7 shows the results presented in the sample details view D when the numbers 46, 47, and 48 in view C are clicked respectively.

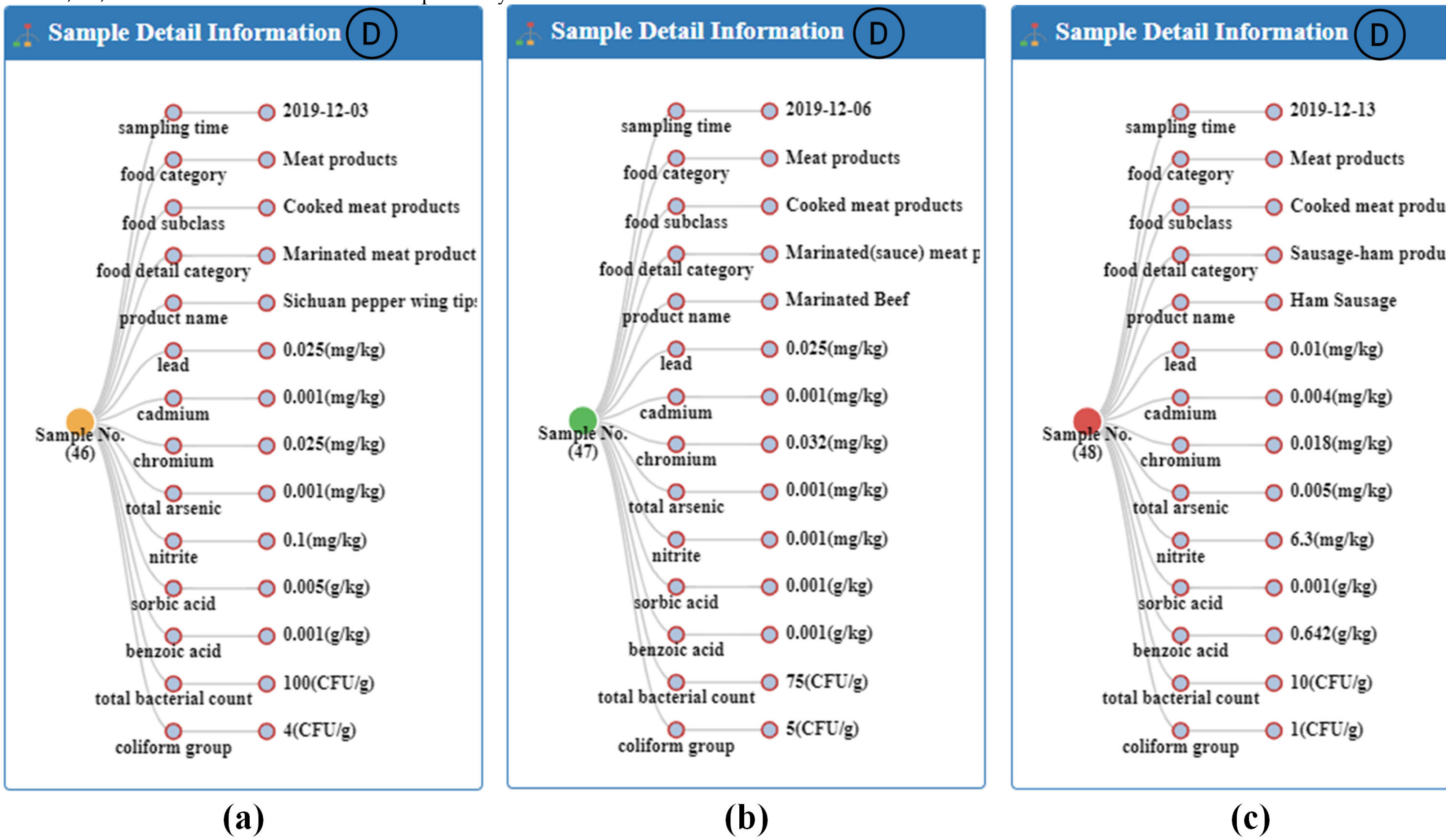

Figure S7. Detail information of Sample No. 46, 47, 48 shown in View D.

4. The proportion of samples by risk levels in view G mainly presents the uploaded data samples, and the proportion of samples of different risk levels obtained after the prediction of the model in this paper, as shown in Figure S8. Changing the high-risk threshold in view A, and view G also changes, as shown in Figure S9.

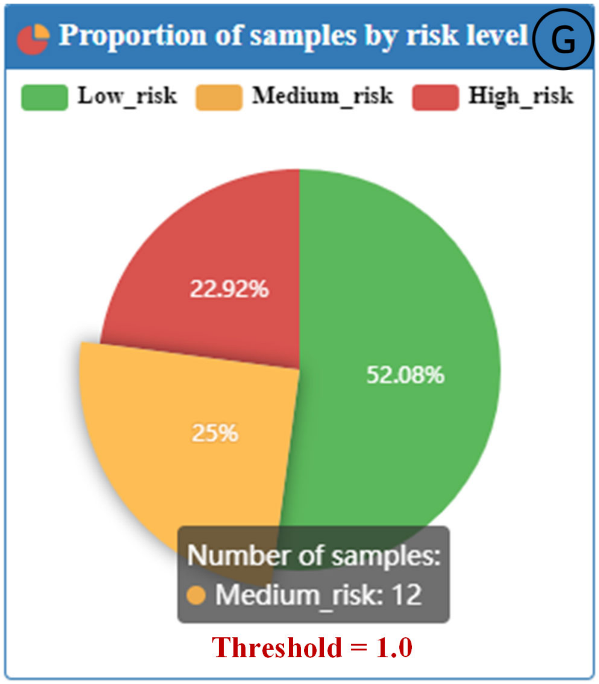

Figure S8. Proportion of samples by risk level shown in View G when the threshold is 1.0.

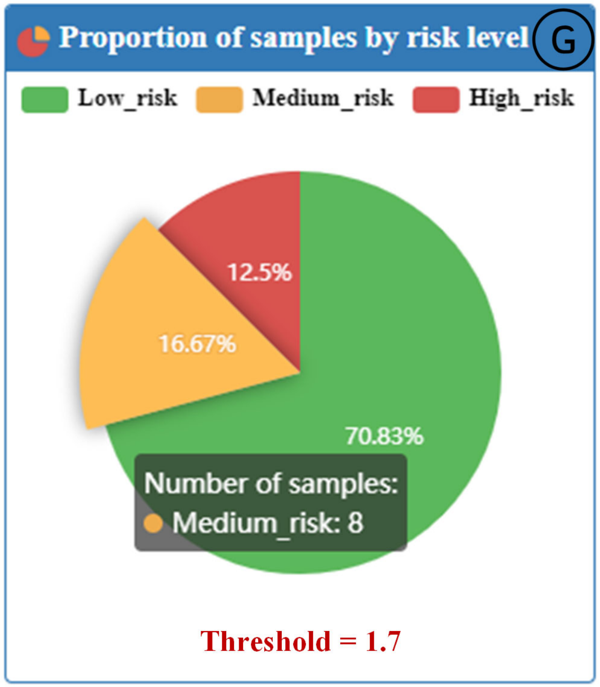

Figure S9. Proportion of samples by risk level shown in View G when the threshold is 1.7.

5. The relative risk of each indicator is presented in the risk composition of samples view F using parallel coordinates, where each axis represents an indicator and each line represents a sample, which crosses each axis. The color channels indicate the level of risk, as shown in Figure S10. When the lines are complicated, the chart can use the choose brush tool to select some of the lines, to increase the contrast between the selected and unselected lines, which can highlight the selected lines and reduce the brightness of the unselected lines, as shown in Figure S11; at the same time, you can also select some of the lines to be presented by the legend at the top of the chart, as shown in Figure S12. This interaction applies to View C. Changing the high-risk threshold in View A When the high-risk threshold is changed, view F is also changed, as shown in Figure S13 and S14.

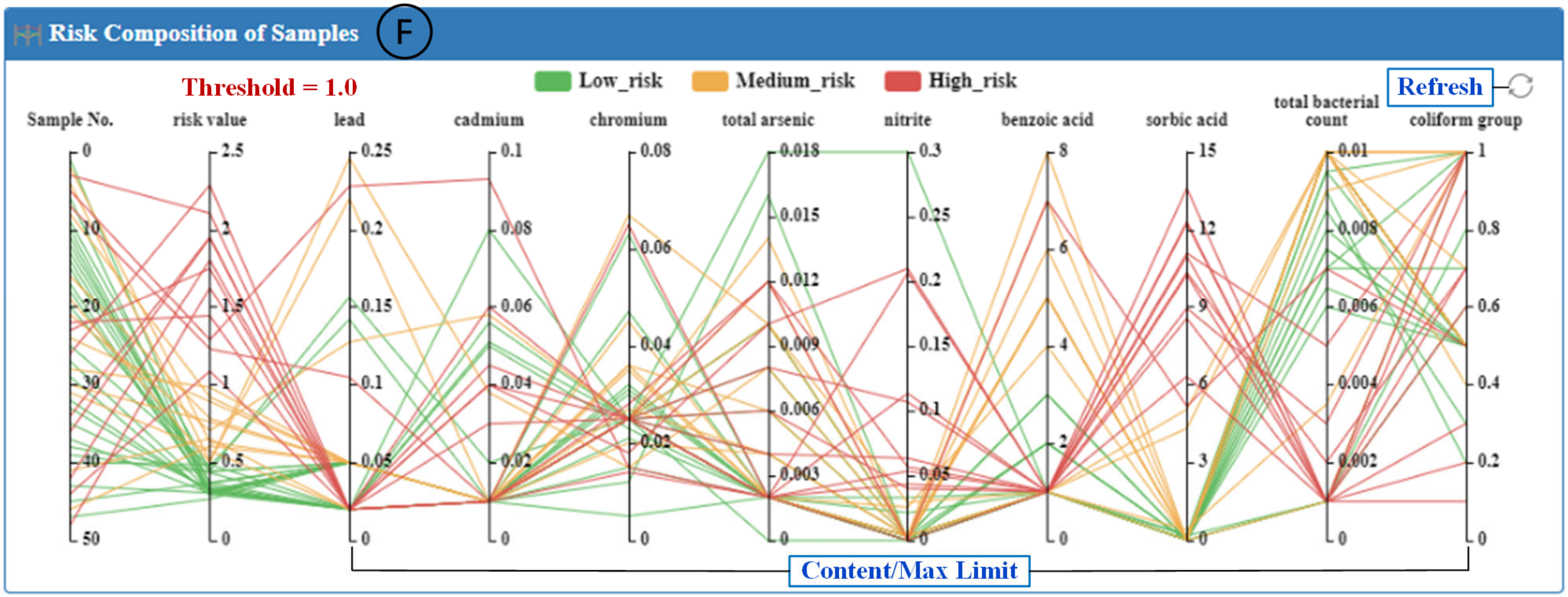

Figure S10. Risk composition of samples shown in View F when the threshold is 1.0.

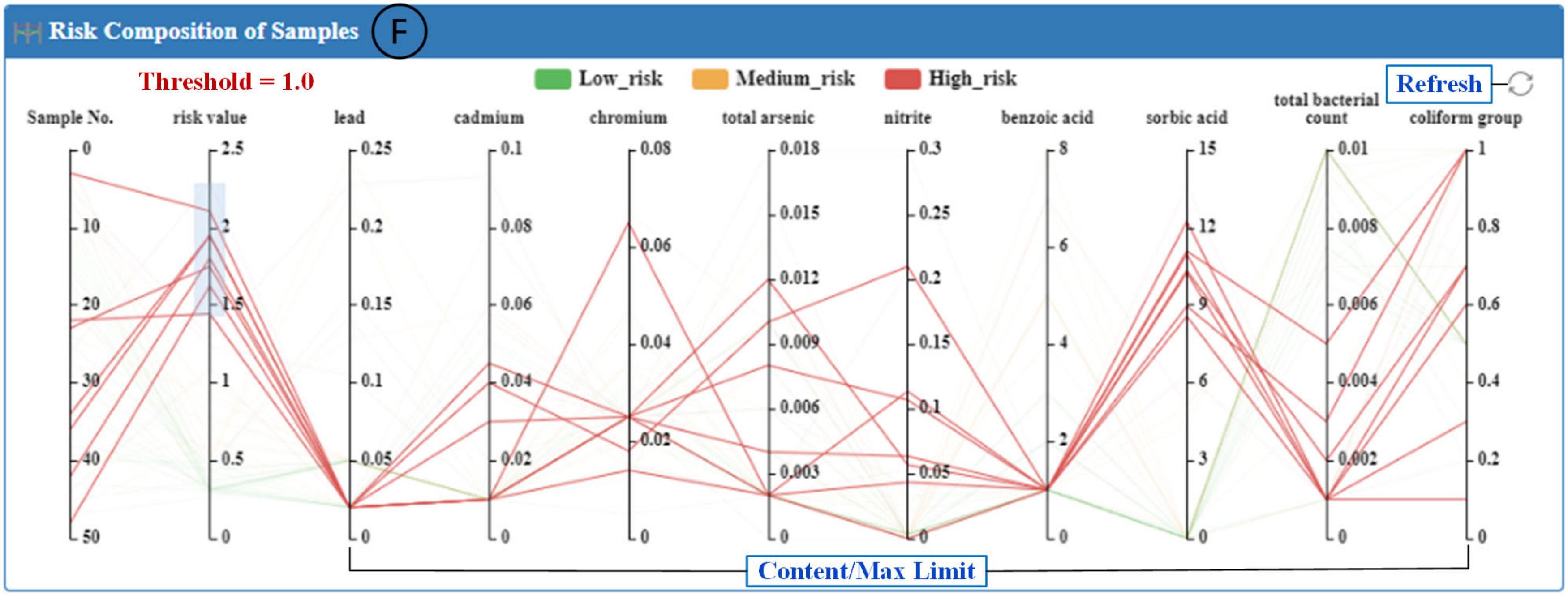

Figure S11. Risk composition of samples after selecting lines greater than 1.5 in the risk value axis using the choose brush selection interaction tool.

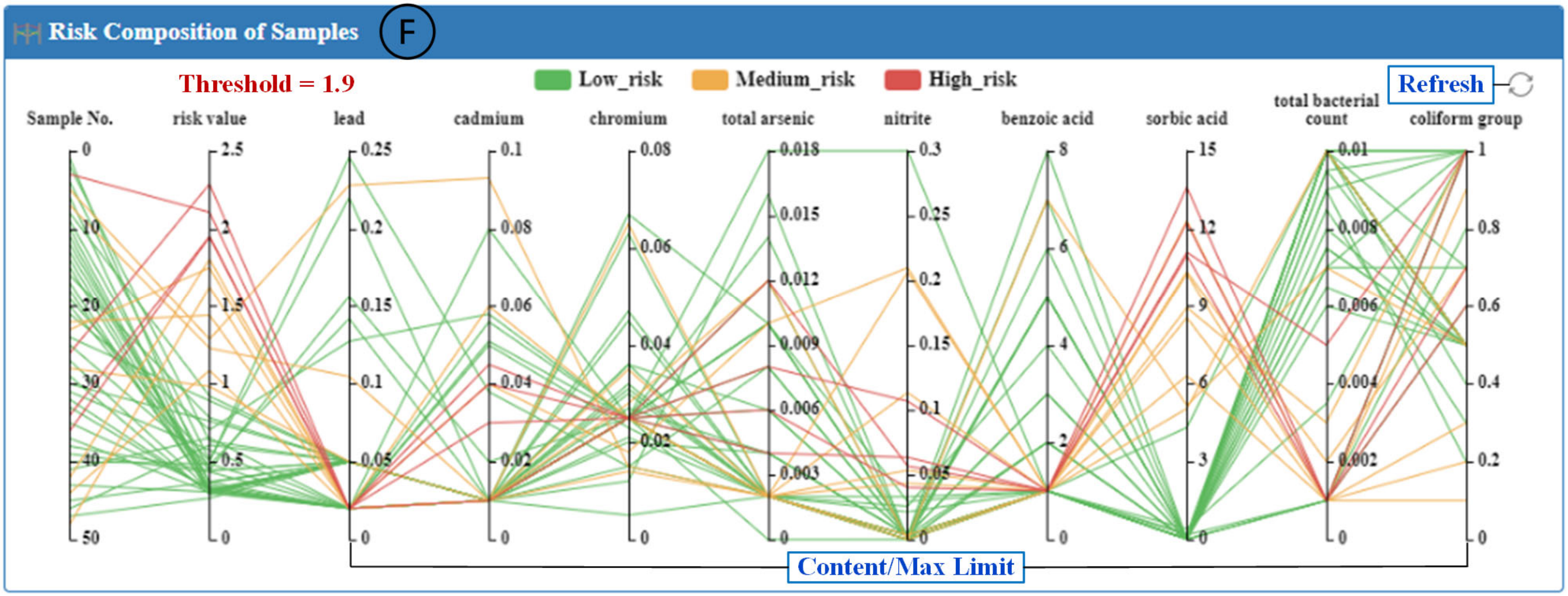

Figure S12. Risk composition of samples shown in View F when the threshold is 1.9.

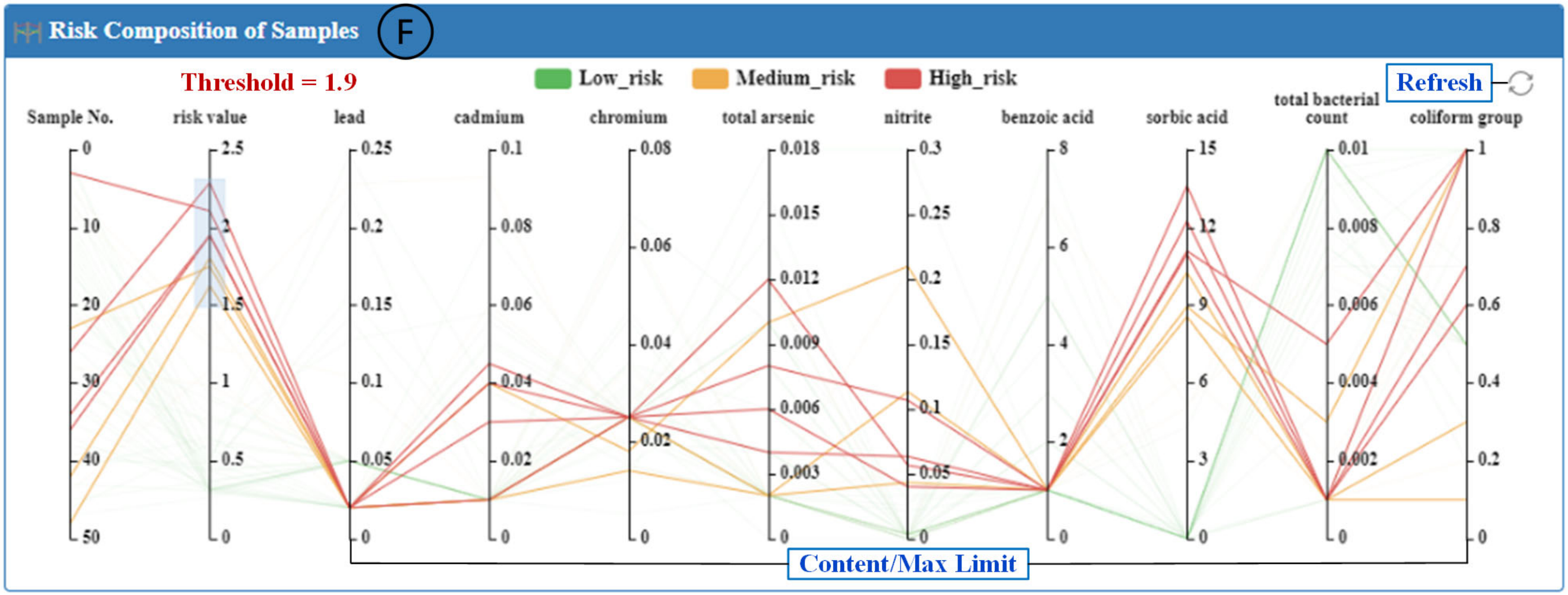

Figure S13. Risk composition of samples after selecting a line greater than 1.5 in the risk value axis using the choose brush interaction tool.

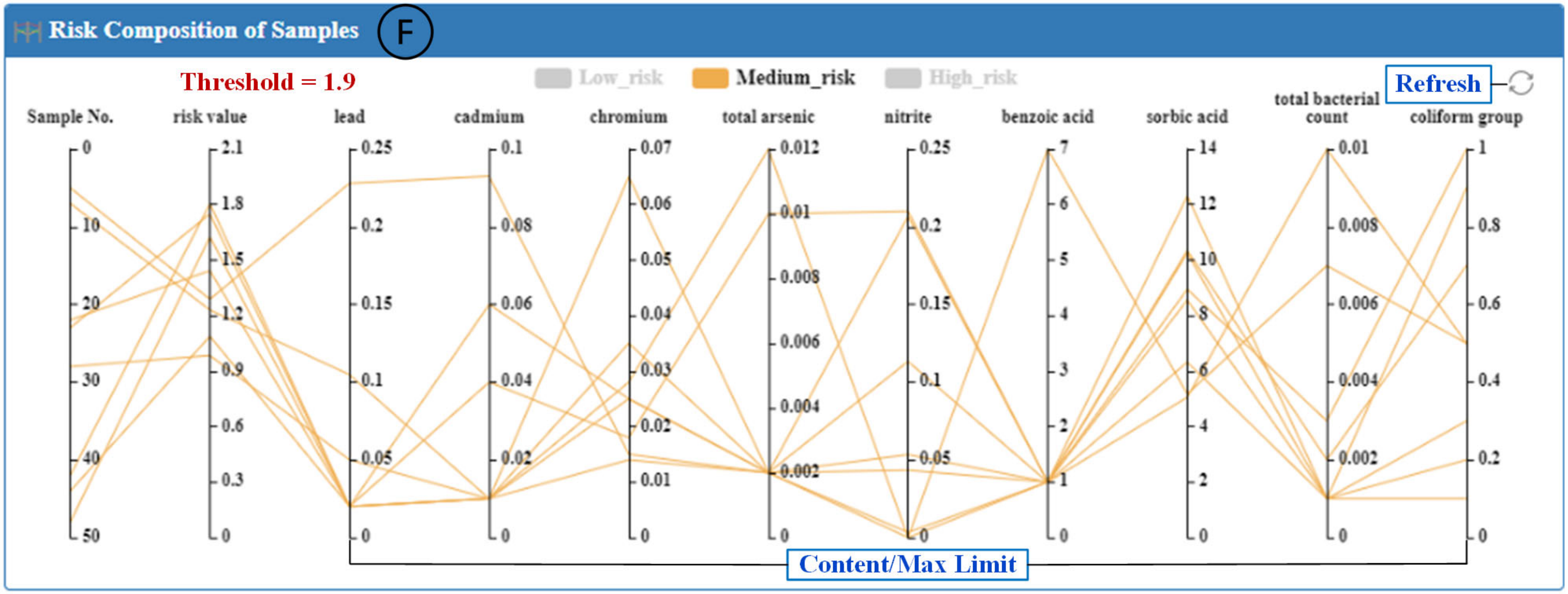

Figure S14. Risk composition of samples after selecting medium risk lines using legend interaction.
